# Experimentation at the interface of science and policy: a multi-case analysis of how policy experiments influence political decision-makers

**DOI:** 10.1007/s11077-017-9276-2

**Published:** 2017-01-28

**Authors:** Belinda McFadgen, Dave Huitema

**Affiliations:** 1 Institute for Environmental Studies (IVM), VU University Amsterdam, De Boelelaan 1085, 1081 HV Amsterdam, The Netherlands; 2 Deputy Department Head Environmental Policy Analysis, Institute for Environmental Studies (IVM), VU University Amsterdam, De Boelelaan 1087, 1081 HV Amsterdam, The Netherlands; 3 Department of Science, Faculty of Management, Science, and Technology, Netherlands Open University, Valkenburgerweg 177, 6419 AT Heerlen, The Netherlands

**Keywords:** Policy experiments, Effectiveness, Institutional design, Science–policy interface

## Abstract

For decades now, scholars have grappled with questions about how knowledge producers can enhance the influence of their knowledge on users and improve policy making. However, little attention has been paid to how policy experiments, a flexible and ex ante method of policy appraisal, obtain influence over political decision-making. To address this gap, an exploratory framework has been developed that facilitates systematic analysis of multiple experiments, allowing hypotheses to be tested regarding how an experiment’s institutional design can influence the views of political decision-makers. Cash’s categories of effectiveness are used to describe an experiment’s conceptual influence; being how credible, salient, and legitimate decision-makers perceive an experiment to be. The hypotheses are tested using 14 experiment cases found relevant to climate adaptation in the Netherlands, with complete survey responses from over 70 respondents. The results show that although, in general, the experiments had medium to high influence on decision-makers, institutional design does have a noticeable impact. Organisers should make choices carefully when designing an experiment, particularly in order to maintain relevance during an experiment’s implementation and to build community acceptance. Suggestions for future research include a comparison of experiment effects with the effects of non-experimental forms of appraisal, such as piloting or ex ante impact assessment.

## Introduction

With so many interests, analyses, and perspectives to be considered when developing policy, how can a decision-maker feel confident about her selection of policy options? One suggestion is to experiment, defined as a flexible, evidence-based approach to policy making that is temporary and reversible (Tassey 2014). Taking an experimental approach to governance forms the cornerstone of Campbell’s “Experimenting Society” where the underlying premise is that policy-relevant knowledge must be created and critiqued using ex ante evaluation and learning (Campbell 1998; see also: Lee 1999; Armitage et al. 2008).

In the policy sciences, discussion focuses on an experiment’s characteristics or suitability for policy making, but empirical analyses of an experiment’s effects are uncommon. Greenberg et al. (2003) conducted the most relevant comparative study when they analysed the political impacts of five US social experiments. Millo and Lezaun (2006) also assessed two regulatory experiments for their political impacts, and Farrelly and Brown (2011) assessed how policy makers perceived the challenges and mechanisms of urban water experiments. Each of these studies conceptualised experimentation and their impacts differently, and only Greenberg et al. (2003) provided a list of factors said to improve the likelihood that an experiment has an influence on policy decisions. Generally, scholarship seldom challenges the foundational assumption that experimentation and learning improve decision-making. It is this research gap we hope to address in this paper.

As an experiment is a venue where science and policy temporarily “engage in elaborate and productive interplay” (Munaretto and Huitema 2012), an understanding of the influence of experimentation on politics can be improved if the focus is on the impact of science–policy interfaces or policy appraisal and evaluation research. Here, empirical work into the relationship between knowledge production and knowledge utilisation, or factors that encourage or limit use of research findings, is more common (e.g. Weiss 1977; Cash et al. 2003; Teirlinck et al. 2012; Koetz et al. 2012; Jordan and Russel 2014). Drawing on these studies, this paper explores hypotheses about how policy experiments influence decision-makers in their policy network.

Models of knowledge utilisation focus on either a conceptual or concrete influence on decision-makers (Greenberg et al. 2003). This paper focuses on conceptual effects and explores to what extent experiments make an impression on decision-makers and how exploring the consequences of research other than its direct application (similar to Weiss’s 1977 analysis of enlightenment). Distinct criteria are used to gauge the effects policy experiments have on decision-makers, including how credible, salient, or legitimate they are perceived to be (Cash et al. 2003). *Credibility* reflects the perceived validity of evidence and arguments, *salience* reflects the extent to which the experiment is seen to be responsive to the needs of decision-makers, and *legitimacy* reflects the perception that the process of producing information and technology has been respectful of stakeholders’ divergent values and beliefs, unbiased in its conduct, and fair in its treatment of views and interests (Cash et al. 2003).

The research setting is the climate adaptation field. The inherent flexibility of experimentation is expected to meet the needs of climate adaptation governance, which has been defined by its uncertainties, controversies, and long-time frame (Massey and Huitema 2013). Experiments have the potential to provide evidence without fully committing to a particular policy action, which may reduce uncertainties and provides flexibility for complexities that arise. The intention of this paper is not to explicitly evaluate these characteristics, but to assess a group of 14 policy experiments (out of a longer list of 147 pilot cases) that provide evidence relevant to climate adaptation, all of which are situated in the Netherlands, where adaptation is a national priority. To assess the experiments, the policy sciences (e.g. Dryzek 1987; Owens et al. 2004; Sanderson 2009) and STS (e.g. Pielke 2007) literature are used to distinguish between three types of experiment: the technocratic, boundary, or advocacy experiment. These ideal types are theoretically constructed as aggregates of governance design choices that differ in the experiment types. The hypotheses suppose that different experiment designs affect how effective experiments are.

Using quantitative research methods in a multi-case study analysis, and drawing on the literature cited above, this paper answers the research question: *what is the relationship between the governance design of a policy experiment and its influence on the policy network?* This question can be broken down into a set of sub-questions, which outline the construction and subsequent testing of the hypotheses:

1. In what ways are political decision-makers influenced by policy experimentation?
2. To what extent does governance design explain how experiments influence political decision-makers?
3. What are the implications of the findings on our understanding of how experiments are used in policy making?

The paper is set out as follows. First, we summarise the different theoretical understandings of policy experimentation before positing a definition of the concept suitable for environmental governance. Next, the dimensions used to measure effectiveness are set out in “Analytical framework” section, which connects experiment design to conceptual influence using hypotheses about how design choices affect the credibility, salience, and legitimacy of the experiments. This is followed by an explanation of data collection and survey methods used. Survey data are analysed to answer the first and second sub-question. Finally, the main findings of and limitations to this research are discussed.

## Policy experimentation: reforms on trial

One of the first scholars to promote the use of experiments for the betterment of society was DT Campbell, who advocated for reliable policy reform using experimental and quasi-experimental approaches in his “Experimenting Society” (Campbell 1969). Campbell was not the first to recognise the value in experimentation for reform, as Dewey back in the 1920s considered democracy “inherently experimental” and policies could be “-experimental in the sense that they will be entertained subject to constant and well-equipped observation of the consequences they entail…”, which is somewhat consistent with Campbell’s notions of exploring new ideas and testing them (Caspary 2000). Supported by similar descriptors of policy development, for example, Lindblom’s (1959) piecemeal implementation of policy in exploratory, incremental steps, the experimenting ideal gained traction and during the following decades experimental interventions were conducted in an attempt to improve economic, health, and education policy, particularly in (but not limited to) the USA and UK (Greenberg et al. 2003). After some years, the appeal of policy experiments diminished, due to the weakened belief in big government and rational planning (Sanderson 2009) but the concept has recently enjoyed a revival, particularly in the realms of environmental governance, including adaptive management (Lee 1999), transition management (Kemp et al. 1998), and climate governance (Hoffman 2011).

These fields understand an experiment as a project temporarily implemented on a limited scale, which injects flexibility into the policy process (Tassey 2014). However, the concept has otherwise stretched with use; in adaptive management, experimentation is expected to provide reliable evidence of whether new management interventions work in complex settings. In transition management, experiments are viewed favourably as niche level projects that are used to diffuse an innovation on a wider scale. In climate governance, experiments are radical innovations, novel improvements that exist outside the political status quo and seek to transform it (Castán Broto and Bulkeley 2013).^1^ Experimentation has recently found a place in the policy innovations literature, where it has been suggested experiments maintain an evaluative function while generating new, innovative policy action (Jordan and Huitema 2014). Following this, a definition of a policy experiment is constructed that captures these characteristics: “*a temporary, controlled field*-*trial of a policy*-*relevant innovation that produces evidence for subsequent policy decisions*”. Ultimately, this definition underlines the assertion that the act of experimentation should be explicit: without appraisal of the intervention’s effects, there is only demonstration of a new initiative, and without innovation, only established ideas are being evaluated.

Various reasons actors choose to conduct an experiment are explored in the literature. Experiments provide substantive evidence of how a proposed policy works in action (Greenberg et al. 2003), but they also have many political uses. They can manipulate the policy process by delaying decision-making, or exploit a window of opportunity and set the policy agenda (Ibid.). Experimentation can bring a broader range of actors and ideas into the policy process by creating “shadow networks” (Olsson et al. 2006), and experiments can build acceptance amongst the local community, which helps us understand what is “appropriate” from their perspective (Greenberg et al. 2003; Millo and Lezaun 2006; Sanderson 2009).

The next section introduces the framework used to assess the relationship between experimentation and effectiveness. Two typologies are developed: one that stems from science–policy evaluation and used to measure an experiment’s conceptual influence (Greenberg et al. 2003; Cash et al. 2003), and one that posits three different experiment designs as “ideal types” [in the sense of Weber (1968)]. Once the typologies are explained, the hypotheses about their relationship are outlined.

## Analytical framework

### Measuring effectiveness

The examination of knowledge production and use in policy making revolves around a number of themes. Venues used to produce knowledge for policy include expert advisory bodies, parliamentary select committees, and policy appraisal settings (Jordan and Russel 2014), of which experiments and pilots are variants.^2^ Experiments as knowledge producers at the science–policy interface are expected to provide decision-makers with evidence of the effects of a policy, which can have concrete or conceptual effects on its audience. In this context, we focus on how an experiment influences a policy actor’s mind set, and a conceptual utilisation process described as the gradual sedimentation of ideas into a policy network (Weiss 1977). This focus contrasts with concrete utilisation, where research findings are found to directly influence specific policy decisions (Greenberg et al. 2003) (also known as the knowledge-driven model in Weiss 1977). Although understanding the direct effects of knowledge on policy decisions is valuable, the number of interacting variables to be considered means demonstrating any impact on actual decisions would be difficult (Turnpenny et al. 2014). Measuring the perspectives of decision-makers, in contrast, is straightforward in comparison and broadens understanding of how experiments influence policy making. Whether a decision-maker uses experimental evidence in their decisions may depend on how favourably they perceive the experiment.

To assess conceptual influence, we draw on criteria regularly used to assess the effectiveness of a science–policy interface—how credible, salient, and legitimate an interface is perceived to be (Cash et al. 2003). The Cash typology is regularly used to assess the science–policy interface and is similar to the criteria that Weiss (1977) used to assess enlightenment experienced by decision-makers (being the perceived technical quality, the relevance of research to policy, and the political acceptability of the research). Credibility refers to the degree to which policy makers consider the experiment authoritative and believable, and to the degree in which they trust the outcomes. It includes credibility of both the knowledge production processes and of the knowledge holders (Saarki et al. 2014). Salience refers to the perceived relevance of the experiment by decision-makers at a certain moment in time. It makes us aware of the relationship between expert knowledge and decision-making, emphasising that credibility alone is not going to improve political decisions (Cash et al. 2003). The third criterion is legitimacy, which reflects the perception that the production of information has been respectful of stakeholders’ divergent values and beliefs, unbiased in its conduct, and fair in its treatment of views and interests. Legitimacy rests on how the choice was made to involve some actors and not others, and how information was produced and disseminated (Ibid.). The three criteria are summarised below (Table 1).

**Table 1:** Three criteria measuring effectiveness, defined by Cash et al. (2003)

Effectiveness criteria	Definition
Credible	Authoritative, of high quality, with trustworthy outcomes
Salient	Relevant to policy at a certain moment in time
Legitimate	A process that fully incorporates the values, concerns, and perspectives of different actors

### Typology of experiments

Studies have analysed experiments in terms of their characteristics (van der Heijden 2014), purpose (Ettelt et al. 2015), and implications for policy (Greenberg et al. 2003). Here, experiments are assessed in terms of how the organiser “sets” the experiment’s institutional rules, as described in the Institutional Analysis and Development Framework developed by Elinor Ostrom (2005). Ostrom uses the rules to describe an action situation, and they determine who is involved and who is excluded (boundary rules), how responsibilities are distributed (choice rules), what types of information are distributed, how regularly, and to whom (information rules), the extent of buy-in by participants (pay-off rules), and how decisions are made (aggregation rules).

How these rules are set can be understood as design choices made by an experiment’s organiser. To facilitate empirical investigation, differences in the settings of each of the rules can be aggregated into three different types of experiment: *technocratic, boundary*, and *advocacy* types. Real-world examples can then be approximated against these types (Weber 1968; Dryzek 1987). Typical diametric technocratic and interpretive approaches to policy analysis (Owens et al. 2004) provide a basis for distinguishing such types. The typology is also informed by a model of the science–policy interface that classifies different roles of science (Pielke Jr. 2007): science as arbiter, issue advocate, or an honest broker of policy options. The sections below summarise the rule settings for each ideal type (rule settings for each experiment type are given in detail in “Appendix 1”).

#### Technocratic experiment

The technocratic policy experiment resembles the technical–rational model of knowledge production, where an expert elite generates scientific knowledge for policy decisions (Owens et al. 2004). It produces scientific information with little or no connection to the policy process until the end, when the results are presented to decision-makers. The experiment thus plays a supposedly objective and disconnected role in politics as “science arbiter” (Pielke Jr. 2007). Knowledge is produced and verified through processes acceptable to the involved scientific community, with fact finding occurring within the parameters of the goals previously set. This arrangement reinforces the view that science is independent of politics (Koetz et al. 2012).

#### Boundary experiment

A boundary policy experiment provides an opportunity for actors—state and non-state—to gain access to and possibly influence policy making. The boundary experiment is initiated by a collaboration of actors, and the production of scientific knowledge is supplemented by multiple knowledge systems—relevant contextual, lay, and traditional forms of knowledge, which are considered of value (Koetz et al. 2012). The experiment’s role in policy making resembles the “honest broker of policy alternatives” (Pielke 2007), where it engages with the policy process and develops policy solutions in accordance with multiple value perspectives. It is expected that the engagement results in participants appreciating the different ways the problem can be understood, and in turn designing and testing a mutually beneficial solution (Lejano and Ingram 2009).

#### Advocacy experiment

By choosing to design their experiment as an advocacy type, an organiser indicates that they have a predefined problem definition and are not open to alternative interpretations. They intend to use the experiment to encourage action in a particular policy direction and to soften objections (compare the “(stealth) advocate” role in Pielke 2007). An advocacy experiment is generally organised by policy makers and includes dominant, traditional actors in coalition. Different actor types might be represented, but they agree with the problem conception and those with contrasting expectations (“outsiders”) are barred from gaining access (Owens et al. 2004). Those in charge retain power and control over design, monitoring, and evaluation procedures, reinforcing the existing structures of power.

To summarise, the three experiment types each represent an aggregate of different rule settings with divergent configurations of information, power distributions, and participants. Individual rule settings could be analysed as independent variables in themselves, but it is not the focus of this analysis (see Leach et al. 2014 for an assessment of how individual design variables affect learning outcomes). The following section outlines how the (conceptually derived) expectations of how the types might produce different effects on decision-makers.

### Experiment design and effectiveness

The literature suggests several factors that could influence credibility, saliency, and legitimacy. Based on these factors, three hypotheses are built that connect the design of experiments to these criteria.

#### **H1**

If an experiment has a technocratic design, it will be considered highly credible and moderately legitimate, but not salient.

For the technocratic type, due to its emphasis on independent scientific methods and expertise the experiment is expected to be considered highly credible (Cash et al. 2003; Owens et al. 2004). These experiments maintain a transparent process and reporting of scientific findings—including uncertainties and limitations, which also boosts credibility (Saarki et al. 2014). Separating the participants (expert actors) from policy makers and excluding discussion on different perspectives means, the experiment is less likely to resonate with the needs of policy makers, reducing the possibility of the project being considered salient. The funding for the experiment is likely to be from organisations with a purely scientific interest, which care more about scientific publications than about policy relevance. Finally, the closed character of the technocratic type reduces its legitimacy because the research question, data gathering process, and report writing have not involved stakeholder groups or ordinary citizens and might not address arguments they consider important (Millo and Lezaun 2006); however, this loss of legitimacy is tempered by the experiment’s openness and transparency.

#### **H2**

If an experiment has a boundary design, it will be considered highly legitimate yet moderately credible and salient.

In a boundary experiment, wide boundary settings ensure that non-state actors have access to policy making where they can influence how a public policy problem is solved (Dryzek 1987). This may result in the experiment being perceived as very legitimate, as the inclusion of different perspectives increases the chance that the evidence resonates with societal needs (Hegger et al. 2012). Boundary experiments are the only type that allow actors to enter the process on their own volition, which improves their legitimacy compared to the other two types. Moreover, open and transparent information transmission between participants allows for the “extended peer review” of the experiment by a range of actors (Funtowicz and Ravetz, 1993), rendering the information produced more legitimate (Ibid.). The inclusion of different knowledge types will distract from the independent and reliable knowledge produced, so a lower perception of credibility than for the technocratic type is expected. Including a range of actors may ensure salience, although increased inclusiveness can have negative effects because it may mean issues are reframed that make an experiment irrelevant (Cash et al. 2003). Nevertheless, a boundary experiment will strengthen linkages between knowledge production and users and increase the probability that the experiment will be designed around the best question for policy (Saarki et al. 2014).

#### **H3**

If an experiment has an advocacy design, it will be considered highly salient but not very credible or legitimate.

Finally, in regard to the expected impacts of an advocacy experiment, credibility is undermined by including policy and non-state actors in the experiment along with expert actors, which dilutes the validity of scientific knowledge with the production of practical knowledge. Moreover, if it is noticed, selective information distribution and a lack of transparency reduce the experiment’s perceived reliability. In the attempt to show there is support for a particular proposal, the organiser blocks participation by critical actors and thereby undermines their concerns, reducing fairness and the perceived legitimacy of the project. However, salience may be high because of the presence of dominant policy actors, which helps when the experiment is used to keep a policy idea alive (Greenberg et al. 2003), and outcomes are presented when the time is right—carefully gauged and engineered by the policy actors involved.

Table 2 summarises the expectations sketched above into three tentative hypotheses.

**Table 2:** Expected scores for the three types

	Credible	Salient	Legitimate
H1: technocratic type	High	Low	Medium
H2: boundary type	Medium	Medium	High
H3: advocacy type	Low	Medium/high	Low

The categories relate to the 1–5 scale used for measuring experiment effects: high ≥4; middle = 3–4; low ≤3 (the questions were answered on a scale ranging from: (1) no certainly not; (2) not really; (3) neutral; (4) somewhat; and (5) certainly)

### Intervening variables

Our independent variable (governance design choices made by experiment organisers) is only one possible way to explain variations in an experiment’s effectiveness. There are competing explanations that may explain their impact; for example, what role (if any) the respondent’s organisation had in the experiment. Other relevant factors include what government institution they work for, or the extent they consider the experiment innovative (Weiss 1977). Playing one of these roles might positively bias a decision-maker’s survey responses. These variables are also operationalised and examined in the analysis below. Other intervening variables include the extent of change in the political environment external to the experiment and environmental crises such as flooding events or drought, but these external changes were not controlled for.

## Data and methods

### Case selection

Based on the definition of policy experiment posited earlier, five criteria were used to isolate experiment cases related to adaptation from a broader set of 147 innovative pilot cases^3^ conducted in the Dutch environment sector. Table 3 below sets out the criteria: whether the project was testing for effects; whether it was innovative with uncertain outcomes; whether it had policy relevance; whether there was state involvement; and whether it was relevant to climate adaptation. Eighteen cases met all five criteria.^4^

**Table 3:** Criteria and associated indicators used to identify policy experiments in climate change adaptation in the Netherlands

Criteria	Indicators	Relevance to definition
Testing for real-world effects	In situ intervention with monitoring and evaluation framework	Temporary “controlled” field trial
Innovation	Previously untried policy or management practice	Innovative intervention with uncertain outcomes
Policy relevance	Test of policy concept or approach	Produces evidence for policy decisions
State involvement	Organiser or other participatory role played by an actor employed by state or state agency	
Ecosystem response	Intervention extends across social–ecological system	
Climate change adaptation focus	Exploring new policy concepts to manage sea-level rise, flooding, freshwater availability, and increased drought	

The 18 experiment cases dealt with a range of issues in climate adaptation: from safety against sea-level rise, increased precipitation, water variability, drought, and saltwater intrusion. Climate adaptation is an emerging policy field, and it is notable that most experiments sampled relate to the distribution and quality of water. The cases demonstrate how the Netherlands are taking broader adaptation-related responses—such as land use planning and agriculture—and coupling them to water concerns, e.g. multifunctional land use; private responsibility being taken for the amount of water used (Wolsink 2010).^5^ Ten experiments tested technical innovations (the application of a technical solution on the ecological system to measure its impacts); four experiments tested governance innovations (the application of a governance solution on the social and ecological system); and four experiments trialled both (see “Appendix 2”). The governance innovations tended to be boundary or technocratic experiments, and the combined governance/technical innovations tended to be advocacy experiments. Most technical innovations were technocratic or advocacy experiments. The textbox below summarises one of the experiment cases, De Kerf.^6^

**Table:** 

De Kerf Coastal Management Experiment (1997–2003)	
This experiment was conducted from 1997 to 2003 and examined the implications of dynamic coastal management in the Dutch province of North Holland. The experiment involved cutting through the fore-dune coastal defence to test the possibility of maintaining a defence while letting natural processes restore the dune areas and maintaining ecological values inside the dunes. This required a change in thinking from an exclusive focus on safety to a broader approach that linked safety objectives with nature objectives and recreation, without compromising safety. “Dynamic coastal management” was an innovation in thinking about coastal management in 1990s policy documents from the Ministry of Transport, Public Works and Water Management (V&W, 1990) and the Ministry of Agriculture, Nature and Food Quality (LNV, 1990). The experiment achieved its goals but monitoring of effects was halted by budget cuts. The results had an impact on the “New Delta!” EU programme, and the innovation was adopted as policy in “de Derde kustnota” (third Coastal note)	

### Data collection

This research measured the effect of each experiment on its surrounding “policy network”, which is defined as the public institutions that govern the geographical context of each experiment: the municipality, the water authority, the province, and the ministerial level (where appropriate). A desktop search was conducted to compile lists of all the “decision-makers”—council members and heads of policy departments (relevant to environmental policy) in each state organisation. A list of these actors from each relevant state organisation was compiled for 17 experiments, with an average of 121 people per case (each experiment is situated in a municipality, a province, and a water board). One experiment was omitted due to its being a nationwide experiment. Three experiment cases were located in the same municipality, so the total sample population was 1694.^7^

Council members with portfolios relating to environmental issues were chosen first; then, the remaining decision-makers were listed alphabetically, and every fourth name was chosen from each institution to create a list of 30 people per experiment^8^ (stratified random sampling). In October and November 2014, an online survey (using the platform “Survey Monkey”) was emailed to the identified people (510 individuals). To encourage participation and prove legitimacy of the survey, an endorsement from the President of the Dutch Union of Water Authorities, Mr. Peter Glas, introduced the survey email.

### Survey design

The survey first asked decision-makers whether they had heard of the experiment conducted in their area. If they had not, they were directed to the end of the survey. If they responded yes, the respondents were then asked seven questions to assess credibility (e.g. their perception of data quality, trust in the experts, and standard of the conclusions); nine questions to determine salience (e.g. whether the questions asked were relevant to policy makers’ needs and whether the evidence created an opportunity to renew policy); and five questions to assess legitimacy (e.g. whether the experiment included all relevant parties from the area and whether the goals of the experiment were in line with community values). The effectiveness variables were measured as ordinal variables, and “Appendix 4” lists the full set of questions (translated from Dutch). For each experiment, the answers were then given a total score for the three criteria.

Control questions in the survey asked what institutional affiliation the respondents had and whether they were involved personally in the experiment. The respondents were also asked whether their organisation played a role in the experiment, including initiator (*N* = 24), funder (*N* = 24), stakeholder (*N* = 60), interested party (*N* = 31), or knowledge broker (*N* = 15).^9^ Data were also collected on whether respondents were involved in deciding whether to conduct the experiment, and whether they thought the experiment was innovative.

### Data analysis

The survey results were analysed using statistical data-analysis software (SPSS 21). Basic descriptive statistics (frequency tables, cross-tabulations) are used to ascertain impact scores, ANOVA (analysis of variance) tests are used to assess whether there are significant differences in scores between ideal types,^10^ and Kruskal–Wallis tests are used to assess differences in the questions used to assess each variable. The decision to use both parametric and nonparametric tests depended on the normal distribution of the data, which was normal for the aggregated data and not normal for the individual questions (see “Appendices 4, 5”).

We received 164 responses from the 510 survey requests, which is a response rate of 32.2%. Ninety-six of those who responded (59%) had heard of the experiment conducted in their area. However, the number of fully completed surveys (where answers are given for over half of the questions for each variable) reduces the number of useable surveys to *N* = 74. “Appendix 3” displays these numbers per experiment and also notes the respondents’ institutions for each case: municipality (responded in 10 cases); water authority (16); province (10); ministry (3), showing that the range of institutional response was broad. The ministry respondents were decision-makers at the Department for Waterways and Public Works (Rijkswaterstaat) at the Ministry of Infrastructure and Environment, and they were included if this institution was involved in a case.^11^ Table 4 shows that water authorities were most heavily represented, possibly because of the endorsement by their president and because other institutions find the issues less relevant to their general agenda.

**Table 4:** Extent of responses from individual institutions

Institution	# responses	Percent knew case	Per cent completed survey
Municipality	37 (23%)	57	36
Water Authority	68 (42%)	72	66
Province	54 (33%)	43	38
Ministry	4 (2%)	100	50
Total	163^a^	62	47.5

^a^Three respondents did not give their institutional affiliation

## Results

In this section, the survey results are presented and analysed. After a general summary, results relevant to the hypotheses are explored in turn. This is followed by an assessment of the intervening factors and how they relate to the three impact criteria. Out of the 18 cases, four were omitted,^12^ so the analysis rests on 14 experiments. An assessment of the 14 cases (see “Appendix 1”) found that five experiments met the technocratic definition (*N* = 24 complete responses from decision-makers), three were boundary experiments (*N* = 15), and six were advocacy experiments (*N* = 35). Experiment type is a nominal, dichotomous variable.

### Results for whole sample

Table 5 summarises the scores for each criterion used to measure impact. Credibility scored highest with 4.2, legitimacy scored 3.8, and salience had the lowest score of 3.6. No criterion scored “low” (under 3), indicating that the experiments were generally well regarded. Reliability of aggregating the questions into variables was determined by computing Cronbach’s alpha (*α*) for each set of questions. The questions assessing all three criteria scored well over the .7 needed to justify the aggregation (De Vaus 2002). To determine whether the variables differed across the ideal types, an ANOVA test was performed (Table 6). It revealed significant scores when the responses for each criterion were divided up into types. This result adds weight to the assumption that a difference in scores is attributable to design.^13^

**Table 5:** Summary of respondents’ scores for the credibility, salience, and legitimacy criteria

Variables measuring effectiveness	Nos. of responses	Minimum	Maximum	Mean	S.D.	Cronbach’s alpha
Credibility*	74	2	5	4.2	.64	*α* = .92
Salience*	74	2	5	3.6	.6	*α* = .84
Legitimacy*	74	1	5	3.8	.76	*α* = .88

Cronbach’s alpha scores signify the reliability of aggregating the survey questions into the variables (*α* = .70 is the minimum acceptable score)

* Whether there were significantly different scores between the ideal types

**Table 6:** ANOVA test results for significance between the ideal types

ANOVA					
	Sum of squares	*df*	Mean square	*F*	Sig.
Credibility					
Between groups	2.973	2	1.487	3.920	.025
Within groups	25.028	66	.379		
Total	28.001	68			
Salience					
Between groups	3.674	2	1.837	6.170	.003
Within groups	20.242	68	.298		
Total	23.916	70			
Legitimacy					
Between groups	5.336	2	2.668	5.156	.008
Within groups	33.638	65	.518		
Total	38.975	67			

Next, the hypothesis of each ideal type is assessed. The aggregated score is presented, but to gain deeper understanding of how the experiments were perceived some of the individual questions are also analysed, particularly those that had answers that showed significant differences between the types (visual representations of how the ideal types differed across each variable are found in “Appendix 4”).

### Differences in conceptual influence between the ideal types

#### Technocratic experiments

Recalling the hypothesis set out in Table 1 (repeated in Table 7 below), technocratic experiments are expected to be considered highly credible, somewhat legitimate, and only slightly salient. From the survey, technocratic-type experiments recorded medium levels for each variable, so expectations were met for legitimacy but not met for salience or credibility. Technocratic experiments score slightly lower than expected for credibility and .3 below the mean. Their aggregated score for credibility was significantly lower than that for boundary experiments. Technocratic experiments were seen to ask more ambiguous questions, and confidence in the experts involved was lower than both boundary and advocacy experiments (although the mean score is still high).

**Table 7:** Results for technocratic experiments across the three effectiveness criteria

Technocratic experiment (*N* = 24)	Credibility*	Salience	Legitimacy
Expectation	High (>4)	Low (<3)	Medium (3–4)
Results	3.9 ✗	3.3 ✗	3.5 ✓
Sample mean	4.2	3.6	3.8

* Significance for this criterion in comparison with the other types

Technocratic experiments earned a low–medium score for salience, also .3 below the mean. This is slightly higher than the anticipated low score, although significantly lower than advocacy experiments. In particular, technocratic experiments were seen to be primarily linked to expert interests (and not policy interests) more than the other types. Their questions were considered more likely to lose relevance to policy makers over time, and their results less convertible into policy. Technocratic experiment scored medium for legitimacy, confirming the hypothesis. On all five questions, the technocratic experiments did well, although still significantly lower than boundary experiments, particularly in reference to the degree of openness afforded by the experiment.

#### Boundary experiments

Boundary experiments were estimated to be highly legitimate and somewhat credible and salient, and they met two of the hypothesised scores because they scored higher for credibility than expected (Table 8). The high credibility score was a surprise (being .3 above the mean) with all seven questions being given high scores (see “Appendix 4”). As noted above, boundary experiments were considered significantly more credible than technocratic experiments. The salience score was quite a bit lower but still met the hypothesis. Boundary experiments were seen to produce results more convertible to policy than the others, and they were most successful at communicating their results, so that policy makers can utilise them.

**Table 8:** Results for boundary experiments across the three effectiveness criteria

Boundary (*N* = 15)	Credibility*	Salience	Legitimacy*
Expectation	Medium (3–4)	Medium/high (3.5–4)	High (>4)
Results	4.5 ✗	3.7 ✓	4.3 ✓
Sample mean	4.2	3.6	3.8

* Significance for this criterion in comparison with the other types

As expected, boundary experiments excelled in legitimacy. Here, they scored .5 higher than the average and scored high on four of the five measured factors, with the highest score being for most strongly reflecting the views and priorities of people living in the surrounding area where the experiments were embedded.

#### Advocacy experiments

Finally, advocacy experiments were expected to be considered highly salient and only slightly credible and legitimate (the hypothesis is repeated in Table 9). The results show that the assumptions made were wrong for all three criteria. Advocacy experiments were expected to have low credibility but received a high score that equalled the mean. The highest score related to the perceived reliability of the organisers.

**Table 9:** Results for advocacy experiments across the three effectiveness criteria

Advocacy (*N* = 35)	Credibility	Salience*	Legitimacy
Expectation	Low (<3)	High (>4)	Low (<3)
Results	4.2 ✗	3.8 ✗	3.9 ✗
Sample mean	4.2	3.6	3.8

* Significance for this criterion in comparison with the other types

Salience was expected to be high, and although the advocacy experiments scored higher than the mean for this criterion, it was not as high as expected. Nevertheless, the questions posed by advocacy experiments were seen as more relevant than either boundary or technocratic experiments (“Appendix 4”). They scored lowest for their communication of results, which were judged as unfamiliar to policy makers’ experiences, but all types scored poorly on this aspect of salience. Advocacy experiments were expected to not be seen as particularly legitimate, and here they perform much better than assumed. All the questions received medium scores, and they did better than the technocratic experiments.

#### Relevance of the intervening variables

The ANOVA tests demonstrated that experiment type had an effect on the scores for credibility, salience, and legitimacy. However, other intervening explanations might also be relevant. These variables included what kind of organisation the respondent worked for (municipality, province, water authority) and what sort of role the respondent’s organisation had in the experiment (initiator, financier, stakeholder, knowledge provider). The influence of these variables was examined with a series of Mann–Whitney *U* tests (as they did not all meet the normal distribution assumption). In addition, based on Weiss’s finding that innovative research carried greater, more favourable weight amongst decision-makers (Weiss 1977), the extent to which respondents thought the experiment was innovative^14^ and whether this would have an effect on their scoring of an experiment was tested.

The results showed that a decision-maker from an organisation that initiated an experiment was significantly more likely to evaluate an experiment more positively for all three variables and that a respondent from an organisation that funded an experiment was more likely to evaluate the experiment as credible. For the other roles a respondent’s organisation might have had (stakeholder, knowledge provider) the respondents did not vary significantly in scores. In terms of what organisation a respondent worked for, no significant differences were found. Unfortunately, the innovation data violated assumptions required for ANCOVA (the statistical test used to assess the effects of a covariate in an ANOVA test) so the variable was separately examined using a Spearman rho correlation analysis. The test revealed that there was a moderate correlation between innovation and credibility (*r* = .576), weak correlation with salience (*r* = .340), and a moderate correlation with legitimacy (*r* = .619). This implies some corroboration for Weiss’s hypothesis, in the sense that the more an experiment is considered innovative, the higher its credibility and legitimacy.

## Discussion and conclusions

The purpose of this paper was to gain a better understanding of the relationship between the design of a policy experiment and its impact on the policy network, which was measured by assessing a decision-maker’s perception of how credible, salient, and legitimate they believed an experiment to be. Encouraged by Weiss (1977) and Greenberg et al. (2003), we assessed the conceptual influence of an experiment on decision-makers to develop alternative insights into the use of knowledge in decision-making, which go beyond the more regular assessment of the direct use of an experiment’s results. The research questions outlined in the introduction are now discussed in turn, followed by an assessment of the limits to the analysis and future research suggestions.

Regarding question one, results of the survey confirm that decision-makers are generally aware of and hold favourable impressions of policy experiments. This finding should buoy advocates who value the novel, innovative aspects of experimenting for policy development. However, our focus on conceptual influence does not enhance our understanding of whether experiments directly influence policy decisions, and the literature is not very positive: Greenberg et al. (2003) found no indication that the effects of the evaluated experiment cases had been decisive in the decision to adopt a tested policy. Rather, political reasons were given precedence over experiment evidence. Thus, the question of whether experimenting is worth the time and money is still open to debate.

Our second question asked whether an experiment’s governance design affects perceived levels of credibility, salience, and legitimacy of experiments, and we suggested hypotheses for each experiment type. Hypothesis H1 (technocratic experiments) was partially rejected, with only the legitimacy score being predicted correctly; H2 was partially accepted with our salience and legitimacy scores being correct; and H3 was rejected outright because all three effect variables scored differently to predictions.

The analysis shows that, although we predicted over half the relationships incorrectly, most differences in impact scores can be explained by design, particularly the differences between technocratic experiment scores and the other two types. An elitist design is least effective, with a surprisingly medium score for credibility. To decision-makers, isolating experts from policy and limiting inclusion makes an experiment less credible, not more. This critical perception of technocratic experiments reflects the rejection of the “scientification” of politics, as they generated the least trust in their experts and questions were considered the least clear. These results also highlight an evolution in the literature. At its inception, credible evidence was defined as the perceived scientific adequacy of a science–policy interface’s technical evidence and arguments (Cash et al. 2003). Generalisable, scientific knowledge was considered most plausible and accurate. However, during its theoretical development, credibility has essentially been broadened to include place-based knowledge in science (e.g. Hegger et al. 2012). Our results show, for experiments at least, that when a broad set of actors contribute contextual, practical knowledge, this place-based knowledge improves credibility over scientifically defensible knowledge alone (even with a lack of transparency and poor information distribution between the participants, as found in advocacy experiments).

Another interesting observation is the extent advocacy experiments scored surprisingly well on all three impact variables, particularly legitimacy. This may be because all the advocacy experiments were sited in relatively harmonious contexts, where no critical actors were excluded nor objections voiced about the incumbent power structures. Decision-makers would only know actors in their area were disaffected if anyone actually protested about it. We tentatively suggest that an advocacy design reduces an experiment’s perceived legitimacy only if it is used to test policy options in conflict settings, where other actors oppose the proffered solution.

The third question asked what the implications of the findings are on our understanding of how experiments are used in policy making. The uses noted earlier: to produce evidence, build acceptability, and push/maintain ideas on the policy agenda can all be seen reflected here. Strong credibility illustrates that decision-makers value the way evidence is produced; we have not measured whether they apply the evidence to their own decisions, but we can extrapolate from the findings that experiments are generally seen to produce useful and valid information. A medium score for salience reveals that experiments are perhaps vulnerable to political machinations; the length of time it takes to conduct them means they can lose visibility easily and risk becoming a “time capsule” (Sanderson 2002). Organisers must work hard to keep the issue on the political agenda and maintain relevance (Greenberg et al. 2003). Finally, a medium–high score for legitimacy indicates that experiments can play a strong role in “softening” a community’s stance on change, particularly if a boundary design is used that provides non-state actors with access to the process and gives them some control over it.

In regard to research limitations, systematic survey sampling has its strengths as well as its drawbacks. Setting the minimum at seven responses when 40 people are initially sampled is low and limits the findings; however, due to the difficulty in accessing decision-makers at this level and in these numbers, the results validly increase understanding of to what extent political decision-makers value experiments. Randomly choosing respondents also strengthens the findings for each experiment, although we note the unintended bias towards respondents from the water authorities, perhaps due to the endorsement letter from their chairman, and lack of responses from ministry officials. An in-depth qualitative analysis of a selection of these experiments could explore the broad patterns we identified here more thoroughly and avoid these unfortunate biases.

It was surprising that so many people who initially responded to the survey (almost half) had never heard of the projects. The survey participants were randomly chosen, but decision-makers with environmental portfolios were targeted first, which indicates that experiments are being conducted in jurisdictions where relevant decision-makers are not aware of them. This insight could translate into an interesting research question for scholars interested in experiments: building on Greenberg et al. (2003), what strategies should experiment organisers use to ensure an experiment’s visibility and influence? As noted above, we did not control for some extraneous variables; for example, the extent that a political change or external event could influence how salient an experiment is. If a government that championed a policy innovation is replaced by one that is ambivalent or even hostile towards the change, an experiment could quickly lose visibility and relevance; similarly, if a catastrophic environmental event like a flood or earthquake occurred, then this could push the experiment into the spotlight as a symbol of what’s “being done” to manage the disaster. How important these events are to the success of an experiment could be another question for further research.

Scholars might also analyse differences between the effectiveness of an experiment and other policy appraisal venues, such as pilots or cost–benefit analyses; or how different individual rule settings influence an experiment’s effectiveness. A fourth research path could look to establish whether the ideal type designs could fit particular problem contexts, i.e. as mentioned above that advocacy experiments may maintain legitimacy in more harmonious contexts. This resonates with a discussion in Owens et al. (2004) on the choice of different policy appraisal designs being contingent on a continuum from well-structured problems to severely unstructured ones.

To conclude, the analysis reveals that, on the whole, policy experiments have a positive effect on their relevant policy network. In general, they are seen to be of good quality and produce results that are very credible, moderately salient, and moderately legitimate. That decision-makers find the process of experimentation a largely positive endeavour is a useful finding, but the research exposes the fact experiments are only rarely used to invent and evaluate new policy strategies.^15^ The literature agrees: as a method of developing new policy approaches generally, experimentation is uncommon (Gunderson 1999). If governments do attempt institutional reform, the evaluation of outcomes is lacking (Campbell 1998) or innovation may go undetected, with policy change mostly occurring incrementally within existing programmes where the degree of uncertainty is low (Vedung 1997; Campbell 1998). It is noted from the scoping interviews with initiators that the cost and the uncertainty of risk make it hard for policy makers to accept that political failure is an option (a phenomenon explored by Hood 2007). They stressed the importance of building political and public support for the project before it even enters the experiment phase (a factor not captured in the framework) and being explicit in the fact that if the experiment fails, the costs will be borne by the state. On one hand, it makes sense that policy actors are cautious about innovating and taking risks, since they are spending public money. On the other, the costs of maintaining the status quo may eventually outweigh the costs of trying new ways to keep society’s proverbial head above water. Our results should support and inspire actors who want to use experimentation as a method to assess their future innovations.

## Appendix 1

Based on data from an earlier survey, each case was individually assessed using 15 indicators and subsequently assigned an ideal type. The indicators were developed based on the institutional rules as proposed by Ostrom (2005). Each indicator has three settings, one for each ideal type. The indicators and their specific rule settings are shown below. In order to categorise each experiment, the cases were assessed against each indicator based on a predetermined setting for each type. For each case, out of the 15 indicators one ideal type setting emerged as the most dominant. The case was then labelled that ideal type. For example, out of 15 indicators, experiment 2 scored one for technocratic, nine for boundary, and five for advocacy; thus, it was classified as a boundary experiment. One ideal type emerged as the dominant type for each case although it was uncommon for experiments to have all 15 indicators the same for one type.

**Table:**

Rule types^a^	Design choice indicator	Technocratic experiment	Boundary experiment	Advocacy experiment
Boundary	Actor constellation	Expert actors	All actor types involved	Predominantly members of an advocacy coalition
	Access to experiment	Required	Those requesting involvement	Those invited by initiator
	Criteria for new participants	Expert actors	Those with local and/or expert knowledge	Those in support or who will build support for experiment
Position	Initiator role	Expert actors	Collaborators	Policy actors
	Use of facilitator	None	Yes (and neutral)	If yes, then with/for core members only
Information	Contribution to goals	None—already set by policy makers	By all actors	By actors who are in agreement
	Lay knowledge acknowledged/accepted	No	Yes, to a large degree	Yes, but not solely
	Scientific knowledge acknowledged/accepted	Exclusively	As one of many inputs	Only if from scientists within the coalition
	Information transmission	Information for majority	Information for all participants	Information for minority
	Opportunities for personal contact between scientists and policy makers	Few	Frequent	Very frequent but only within the group
	Outsiders informed of progress	Occasionally	Frequently	Rarely
Choice	Authority at decision nodes	Expert initiators	Participants share power	Policy initiators
	Variation in authority	Most actors have advisory role	Most actors have decision role	Most actors have no authority
Pay-off	How costs distributed	Minimal buy-in	Buy-in	No buy-in
Aggregation	How decisions are made	By experts in majority (in line with scientific methods)	Everyone by consensus (on basis of deliberation)	Policy actor by majority (on basis of shared principles)

^a^cf. Ostrom (2005)

## Appendix 2

Breakdown of experiment case into policy issues and solutions, followed by two examples

**Table:**

Exp.	Policy issue/type of problem	(New) policy concept/how tested
Exp 1	Coastal management/sea-level rise	Building with nature. Technical innovation
Exp 2	Coastal management/sea-level rise	Building with nature. Technical innovation
Exp 3	Dike management/river-level rise	Optimal spatial planning. Technical innovation
Exp 4	Freshwater availability/decline in freshwater availability	Shared responsibility. Technical and governance innovation (control site)
Exp 5	Water variability/increase in flooding or drought risk	Multifunctional land use/shared responsibility. Technical and governance innovation
Exp 6	Freshwater availability/decline in freshwater availability	Water husbandry/shared responsibility. Technical innovation
Exp 7	Water variability/increase in flooding or drought risk	Shared responsibility. Technical and governance innovation
Exp 8	Water variability	Saltwater–freshwater transitions. Governance innovation
Exp 9	Water variability/increase in flooding or drought risk	Multifunctional land use. Technical innovation
Exp 10	Coastal management/sea-level rise	Climate buffers. Technical innovation
Exp 11	Coastal management/sea-level rise	Dynamic coastal management. Technical innovation
Exp 12	Dike management/river-level rise	Pest management. Governance innovation (control site)
Exp 13	Water variability/increase in flooding or drought risk	Dynamic level management. Governance innovation
Exp 14	Water variability/increase in flooding or drought risk	Flexible groundwater irrigation. Governance innovation
Exp 15	Water variability/increase in flooding or drought risk	Multifunctional land use/shared responsibility. Technical and governance innovation
Exp 16	Water variability/increase in flooding or drought risk	Multifunctional land use. Technical innovation
Exp 17	Coastal management/sea-level rise	Building with nature. Technical innovation
Exp 18	Water variability/increase in flooding or drought risk	Multifunctional land use. Technical innovation

## Appendix 3

The table notes survey responses for each experiment (Mun. = municipality; W.A. = water authority; Prov. = province; Min. = Ministry for Environment)

**Table:**

Exp # and ideal type	# Initial responses	# Know of the case	# Complete responses	Municipality	W.A.	Prov.	Min.
1 (T)	9	5	4	✗	✗	✗	✗
2 (B)	9	9	6		✗	✗	
3 (A)	10	4	4		✗		
4 (A)	8	6	5	✗	✗		
5 (A)	11	9	9	✗	✗	✗	
6 (T)	9	5	4		✗	✗	
7 (B)	11	4	*1*		✗		
8 (B)	4	1	*0*			✗	
9 (A)	11	2	*2*	✗	✗		
10 (B)	11	8	4	✗	✗		✗
11 (T)	7	7	7	✗	✗	✗	✗
12 (T)	12	8	5	✗	✗	✗	
13 (B)	10	5	4		✗		
14 (A)	7	6	5	✗	✗	✗	
15 (A)	9	8	5	✗	✗		
16 (A)	10	6	6	✗	✗	✗	
17 (T)	16	4	4		✗	✗	
Total (ave)	164	97 (60%)	75 (48%)	10	16	10	3

## Appendix 4

Survey questions and breakdown of answers into ideal types:

1. Credibility

The questions were answered on a scale ranging from: (1) no certainly not; (2) not really; (3) neutral; (4) somewhat; and (5) certainly.

A: Are the findings reliable enough to base policy decisions on?
B: Are the conclusions of the initiators of the experiment well substantiated?
C: Did the experiment produce reliable data?
D: Did the experiment address a clear question or problem?
E: In your opinion, was the experiment well structured?
F: Was the experiment conducted by reliable parties?
G: Did you have confidence in the ability of the scientists/technical experts who were involved in the experiment?

- Graph to show how the ideal types differ for each question measuring credibility. Average = 4.1:

Kruskal–Wallis tests for significance between indicators:

**Table:**

Indicator	Significance	Decision
A	.352	Retain the null hypothesis
B	.086	Retain the null hypothesis
C	.129	Retain the null hypothesis
D	.009	Reject the null hypothesis
E	.078	Retain the null hypothesis
F	.777	Retain the null hypothesis
G	.008	Reject the null hypothesis

2. Salience

A: The experiment delayed rather than accelerated policy development
B: Communication of the results of the experiment did not meet the experiences of policy makers
C: At first the experiment addressed questions raised by policy makers, but now there are new questions
D: The experiment is primarily linked to the interest of experts and not policy makers
E: The experiment produced results that can be converted directly into policy
F: The results of the experiment were communicated clearly, so that policy makers can utilise them
G: The experiment provides substantial opportunities for policy renewal
H: The experiment fits in well with the knowledge gaps that exist amongst policymakers
I: The experiment is about a matter of public interest in my area

- Graph to show how the ideal types differ for each statement measuring salience. Average = 3.6:

Kruskal–Wallis tests for significance between indicators:

**Table:**

Indicator	Significance	Decision
A	.210	Retain the null hypothesis
B	.218	Retain the null hypothesis
C	.008	Reject the null hypothesis
D	.002	Reject the null hypothesis
E	.027	Reject the null hypothesis
F	.560	Retain the null hypothesis
G	.171	Retain the null hypothesis
H	.270	Retain the null hypothesis
I	.040	Reject the null hypothesis

3. Legitimacy

A: Are the goals of the experiment representative of the values existing in the surrounding community?
B: Do you think the organisers of the experiment ensured a sufficient degree of openness?
C: Does the new approach tested in the experiment reflect the views and priorities of people that live in the area?
D: Are the perspectives of participants in the experiment treated with respect?
E: According to you, did the organisers involve in the experiment all parties with affected interests? (Open question respondents were directed to if they answered E negatively): You indicated in the previous question that not all parties with interests in the experiment were involved. Can you specify which parties or interests were overlooked?

Graph to show how the ideal types differ from each question measuring legitimacy. Average = 3.8:

Kruskal–Wallis tests for significance between indicators:

**Table:**

Indicator	Significance	Decision
A	.353	Retain the null hypothesis
B	.013	Reject the null hypothesis
C	.022	Reject the null hypothesis
D	.004	Reject the null hypothesis
E	.012	Reject the null hypothesis

## Appendix 5

Statistics for each impact indicator (dependent variable).

1. Levene’s test of variance results

**Table:** Test of homogeneity of variances

	Levene statistic	*df*1	*df*2	Sig.
Credibility	1.724	2	66	.186
Salience	.516	2	68	.599
Legitimacy	.838	2	65	.437

2. Tukey post-test

**Table:** Multiple comparisons

Tukey HSD					
Dependent variable	Mean difference (I − J)	Std. error	Sig.	95% Confidence interval	
				Lower bound	Upper bound
Credibility					
Technocratic					
Boundary	−.59793*	.21368	.018	−1.1103	−.0856
Advocacy	−.23082	.16727	.357	−.6319	.1702
Boundary					
Technocratic	.59793*	.21368	.018	.0856	1.1103
Advocacy	.36711	.20165	.171	−.1164	.8506
Advocacy					
Technocratic	.23082	.16727	.357	−.1702	.6319
Boundary	−.36711	.20165	.171	−.8506	.1164
Salience					
Technocratic					
Boundary	−.39981	.18495	.085	−.8430	.0433
Advocacy	−.51019*	.14730	.003	−.8631	−.1572
Boundary					
Technocratic	.39981	.18495	.085	−.0433	.8430
Advocacy	−.11038	.17326	.800	−.5255	.3048
Advocacy					
Technocratic	.51019*	.14730	.003	.1572	.8631
Boundary	.11038	.17326	.800	−.3048	.5255
Legitimacy					
Technocratic					
Boundary	−.79429*	.24773	.006	−1.3885	−.2001
Advocacy	−.31003	.19559	.259	−.7792	.1591
Boundary					
Technocratic	.79429*	.24773	.006	.2001	1.3885
Advocacy	.48427	.23770	.111	−.0859	1.0544
Advocacy					
Technocratic	.31003	.19559	.259	−.1591	.7792
Boundary	−.48427	.23770	.111	−1.0544	.0859

* The mean difference is significant at the .05 level

## Footnotes

1 Ansell and Bartenberger (2016) recently captured these different understandings with their typology of controlled, Darwinian, and generative experiments.

2 Jordan and Turnpenny (2015) define policy formulation venues as: “institutional locations, both within and outside governmental settings, in which policy formulation tasks are performed, with the aim of informing the design, content and effects of policy making activities”, which fits the *modus operandi* of experiments well.

3 For 9 months in 2013, two people searched for cases using phrases such as *test pilots, innovation, experiment, proef, onderzoek, *and* pilot*, on programme websites, ministry, province and water authority websites, and in scoping interviews. Projects that were deemed irrelevant included product testing, concept pilots, modelling projects, and reapplications of the initial experiment.

4 The cases have different spatial and temporal scales and deal with different problems; however, they are comparable due to their meeting the stringent conditions. The start dates of the experiments range between 1997 and January 2013, and seven are ongoing as of June 2015. Cases were included if they have had at least 2 years’ implementation or an interim evaluation (following Van der Heijden 2015). Experiment names are not used here to honour confidentiality agreements with participants.

5 Examples of innovative policy concepts being tested include multifunctional land use, which combines flood reduction and nature management; dynamic coastal management and building with nature, which use natural processes to reduce flood risk; and water husbandry, which encourages farmers to close the water cycle and be self-sufficient with the water they have.

6
https://publicwiki.deltares.nl/display/KvdNLK/Derde+Kustnota?preview=/34799636/34865271/3e%20kustnota.pdf.

7 Fourteen experiment cases were multiplied by 121, instead of 17, as three were located in the same municipality and therefore had the same list of decision-makers.

8 This sampling was done by arranging the names from each institution in alphabetical order and choosing every third person. Each person (*N* = 510) was then emailed the survey link. If, after prompting, there were too few responses, then a second list of people was compiled. The initial email was followed by two reminders. A minimum of four full responses for each case was considered sufficient.

9 This question was not compulsory and more than one answer was possible.

10 Here we use ANOVA in line with established practice for measuring the means amongst three or more variables.

11 The decision to only include RWS decision-makers if the organisation was involved in the experiment was based on the observation that although RWS have regional offices, they are staffed by only a few people, who would have been inundated with survey requests had each case been required to include RWS responses.

12 One experiment was omitted due to it being a national experiment and therefore relevant to all institutions throughout the country, making the collection of survey data from all relevant decision-makers overly time-consuming. Another case was removed from the group because of a lack of responses. Two further cases were also removed because, despite a high number of responses, these political actors had not heard of the experiment in their jurisdiction (see “Appendix 3”).

13 See “Appendix 5” for homogeneity of variances test and a Tukey post hoc test.

14 Measured on a scale from (1) not at all innovative–(4) very innovative.

15 It is worth reiterating that out of 147 relevant pilots in our inventory, only 18 were deemed experiments according to the definition.

## References

[CR1] Ansell CK, Bartenberger M (2016). Varieties of experimentalism. Ecological Economics.

[CR2] Armitage D, Marschke M, Plummer R (2008). Adaptive co-management and the paradox of learning. Global Environmental Change.

[CR3] Campbell DT (1969). Reforms as experiments. American Psychologist.

[CR4] Campbell DT, Dunn W (1998). The experimenting society. The experimenting society: Essays in honor of Donald T. Campbell. Policy studies review annual.

[CR5] Cash DW, Clark WC, Alcock F, Dickson NM, Eckley N, Guston DH, Jäger J, Mitchell RB (2003). Science and technology for sustainable development special feature: Knowledge systems for sustainable development. Proceedings of the National Academy of Sciences of the United States of America.

[CR6] Caspary WR (2000). Dewey on Democracy.

[CR7] Castán Broto V, Bulkeley H (2013). A survey of urban climate change experiments in 100 cities. Global Environmental Change: Human and Policy Dimensions.

[CR8] De Vaus D (2002). Survey research.

[CR9] Dryzek J (1987). Rational ecology. Environment and political economy.

[CR10] Ettelt S, Mays N, Allen P (2015). The multiple purposes of policy piloting and their consequences: Three examples from National Health and Social Care Policy in England. Journal of Social Policy.

[CR11] Farrelly M, Brown R (2011). Rethinking urban water management: experimentation as a way forward?. Global Environmental Change.

[CR12] Funtowicz SO, Ravetz JR (1993). Science for the post-normal age. Futures.

[CR13] Greenberg D, Linksz D, Mandell M (2003). Social experimentation and public policy making.

[CR14] Gunderson L (1999). Resilience, flexibility and adaptive management—Antidotes for spurious certitude?. Conservation Ecology.

[CR15] Hegger D, Lamers M, Van Zeijl-Rozema A, Dieperink C (2012). Conceptualising joint knowledge production in regional climate change adaptation projects: Success conditions and levers for action. Environmental Science and Policy.

[CR16] Hoffman MJ (2011). Climate governance at the crossroads: Experimenting with a global response.

[CR17] Hood C (2007). What happens when transparency meets blame-avoidance?. Public Management Review.

[CR18] Jordan A, Huitema D (2014). Policy innovation in a changing climate: Sources, patterns and effects. Global Environmental Change.

[CR19] Jordan A, Russel D (2014). Embedding the concept of ecosystem services? The utilisation of ecological knowledge in different policy venues. Environment and Planning C: Government and Policy.

[CR20] Jordan A, Turnpenny J (2015). The tools of policy formulation: Actors, capacities, venues, and effects.

[CR21] Kemp R, Schot J, Hoogma R (1998). Regime shifts to sustainability through processes of niche formation: The approach of strategic niche management. Technology Analysis & Strategic Management.

[CR22] Koetz T, Farrell KN, Bridgewater P (2012). Building better science–policy interfaces for international environmental governance: Assessing potential within the Intergovernmental Platform for Biodiversity and Ecosystem Services. International Environment Agreements..

[CR23] Leach WD, Weible CM, Vince SR, Siddiki SN, Calanni JC (2014). Fostering learning through collaboration: Knowledge acquisition and belief change in Marine Aquaculture Partnerships. Journal of Public Administration Research and Theory.

[CR24] Lee KN (1999). Appraising adaptive management. Conservation Ecology.

[CR25] Lejano RP, Ingram H (2009). Collaborative networks and new ways of knowing. Environmental Science and Policy.

[CR26] Lindblom C (1959). The science of muddling through. Public Administration Review.

[CR27] Massey E, Huitema D (2013). The emergence of climate change adaptation as a policy field: the case of England. Regional Environmental Change.

[CR28] Millo Y, Lezaun J (2006). Regulatory experiments: Genetically modified crops and financial derivatives on trial. Science and Public Policy.

[CR29] Munaretto S, Huitema D (2012). Adaptive co-management in the Venice Lagoon? An analysis of current water and environmental management practices and prospects for change. Ecology and Society.

[CR30] Olsson P, Gunderson LH, Carpenter SR, Ryan P, Lebel L, Folke C, Holling CS (2006). Shooting the rapids: Navigating transitions to adaptive governance of social–ecological systems. Ecology and Society.

[CR31] Ostrom E (2005). Understanding institutional diversity.

[CR32] Owens S, Rayner T, Bina O (2004). New agendas for appraisal: Reflections on theory, practice, and research. Environment and Planning A.

[CR33] Pielke RA (2007). The honest broker: Making sense of science in policy and politics.

[CR34] Saarki S, Niemela J, Tinch R, van den Hove S, Watt A, Young J (2014). Balancing credibility, relevance and legitimacy: A critical assessment of trade-offs in science–policy interfaces. Science and Public Policy.

[CR35] Sanderson I (2002). Evaluation, policy learning and evidence-based policy making. Public Administration.

[CR36] Sanderson I (2009). Intelligent policy making for a complex world: Pragmatism, evidence, and learning. Political Studies.

[CR37] Tassey G (2014). Innovation in innovation policy management: The experimental technology incentives program and the policy experiment. Science and Public Policy.

[CR38] Teirlinck P, Delanghe H, Padilla P, Verbeek A (2012). Closing the policy cycle: Increasing the utilization of evaluation findings in research, technological development and innovation policy design. Science and Public Policy.

[CR39] Turnpenny J, Russel D, Jordan A (2014). The challenge of embedding an ecosystem services approach: Patterns of knowledge utilisation in public policy appraisal. Environment and Planning C: Government and Policy.

[CR40] Van der Heijden J (2015). What ‘works’ in environmental policy-design? Lessons from experiments in the Australian and Dutch building sectors. Journal of Environmental Policy & Planning.

[CR41] Vedung E (1997). Public policy and program evaluation.

[CR42] Weber M (1968). Economy and society: An outline of interpretative sociology.

[CR43] Weiss C (1977). Research for policy’s sake: The enlightenment function of social research. Policy Analysis.

[CR44] Wolsink M (2010). Contested environmental policy infrastructure: Socio-political acceptance of renewable energy, water, and waste facilities. Environmental Impact Assessment Review.

